# Right Ventricular Myocardial Adaptation Assessed by Two-Dimensional Speckle Tracking Echocardiography in Canine Models of Chronic Pulmonary Hypertension

**DOI:** 10.3389/fvets.2021.727155

**Published:** 2021-08-16

**Authors:** Yunosuke Yuchi, Ryohei Suzuki, Haruka Kanno, Takahiro Teshima, Hirotaka Matsumoto, Hidekazu Koyama

**Affiliations:** Laboratory of Veterinary Internal Medicine, Faculty of Veterinary Science, School of Veterinary Medicine, Nippon Veterinary and Life Science University, Musashino, Japan

**Keywords:** dog, right ventricular remodeling, right ventricular strain, right ventricular-arterial coupling, wall stress, myocardial function

## Abstract

**Background:** Pulmonary hypertension (PH) is a life-threatening disease in dogs characterized by an increase in pulmonary arterial pressure (PAP) and/or pulmonary vascular resistance. Right ventricle adapts to its pressure overload through various right ventricular (RV) compensative mechanisms: adaptive and maladaptive remodeling. The former is characterized by concentric hypertrophy and increased compensatory myocardial contractility, whereas the latter is distinguished by eccentric hypertrophy associated with impaired myocardial function.

**Objectives:** To evaluate the RV adaptation associated with the increase of PAP using two-dimensional speckle tracking echocardiography.

**Animals:** Seven experimentally induced PH models.

**Methods:** Dogs were anesthetized and then a pulmonary artery catheter was placed via the right jugular vein. Canine models of PH were induced by the repeated injection of microspheres through the catheter and monitored pulmonary artery pressure. Dogs were performed echocardiography and hemodynamic measurements in a conscious state when baseline and systolic PAP (sPAP) rose to 30, 40, 50 mmHg, and chronic phase. The chronic phase was defined that the sPAP was maintained at 50 mmHg or more for 4 weeks without injection of microspheres.

**Results:** Pulmonary artery to aortic diameter ratio, RV area, end-diastolic RV wall thickness, and RV myocardial performance index were significantly increased in the chronic phase compared with that in the baseline. Tricuspid annular plane systolic excursion was significantly decreased in the chronic phase compared with that in the baseline. The RV longitudinal strain was significantly decreased in the sPAP30 phase, increased in the sPAP40 and sPAP50 phases, and decreased in the chronic phase.

**Conclusions:** Changes in two-dimensional speckle tracking echocardiography-derived RV longitudinal strain might reflect the intrinsic RV myocardial contractility during the PH progression, which could not be detected by conventional echocardiographic parameters.

## Introduction

Pulmonary hypertension (PH), a life-threatening disease in dogs, is characterized by increased pulmonary arterial pressure (PAP, normal range: systolic PAP; 15–25 mmHg, mean PAP; 10–15 mmHg, and diastolic PAP; 5–10 mmHg) and/or pulmonary vascular resistance (1, 2). The disease would be caused by various diseases in dogs, including pulmonary arterial disease, left heart disease, respiratory disease, hypoxia, pulmonary embolic disease, parasitic disease, or some combination of these (1). Recent studies have reported that PH was one of the risk factors for the worse outcome especially in dogs with myxomatous mitral valve disease and respiratory disease/hypoxia (3, 4). Considering the structural characteristics of the right ventricle, the right ventricular (RV) pressure overload would critically impact RV function and cardiac output (5). In humans, to compensate for low cardiac output due to increased PAP, the right ventricle responds through two compensatory mechanisms: adaptive and maladaptive remodeling (6–9). The former is characterized by concentric hypertrophy and increased compensatory myocardial contractility, whereas the latter is distinguished by eccentric hypertrophy associated with impaired myocardial function. Therefore, to estimate the progression of PH, the change in RV myocardial function and remodeling associated with increasing RV pressure overload must be evaluated.

Currently, various echocardiographic variables are used as clinical, non-invasive tools to assess RV function in veterinary medicine; specifically, two-dimensional speckle tracking echocardiography (2D-STE) enables quantitative, non-invasive assessment of the intrinsic RV myocardial function (10–12). However, studies that have assessed the relationship between invasively measured PAP and echocardiographic variables for RV function in the same individuals are limited (13, 14). Furthermore, almost all these studies have evaluated the association in the acute phase of RV pressure overload in anesthetized dogs (13, 14), although in majority of the cases, PH runs a chronic course and various RV adaptations are exhibited.

We hypothesized that 2D-STE indices would reflect the changes in RV function associated with RV adaptation, and there would be differences in RV function between the acute and chronic phase of RV pressure overload. This study aimed to assess RV morphology and function associated with the increase in PAP during the process of creating model dogs with chronic PH.

## Materials and Methods

Our prospective, experimental study consisted of procedures that were performed in accordance with the Guide for Institutional Laboratory Animal Care and Use in Nippon Veterinary and Life Science University and was approved by the ethical committee for laboratory animal use of the Nippon Veterinary and Life Science University, Japan (approval number: 2019S-56).

### Animals

Seven laboratory male beagles (body weight: 9.1 ± 1.5 kg, age: 1.0 ± 0.2 years) were used in this study. All dogs were determined to be healthy based on a complete physical examination, blood tests, thoracic and abdominal radiography, transthoracic and abdominal ultrasonography, and oscillometric method-derived blood pressure measurement.

### Study Preparation

The study dogs were administered butorphanol tartrate (0.2 mg/kg, IV) (Meiji Seika Pharma Co. Ltd., Tokyo, Japan), midazolam hydrochloride (0.2 mg/kg, IV) (Maruishi Pharmaceutical. Co., Ltd., Osaka, Japan), heparin sodium (100 IU/kg, IV) (AY Pharmaceuticals Co. Ltd., Tokyo, Japan), and cefazolin sodium hydrate (20 mg/kg, IV) (LTL Pharma Co. Ltd., Tokyo, Japan) as pre-anesthetic medication. They were then anesthetized intravenously with propofol (Nichi-Iko Pharmaceutical Co., Ltd., Toyama, Japan), maintained with 1.5–2.0% isoflurane (Mylan Seiyaku Ltd., Osaka, Japan) mixed with 100% oxygen. The end-tidal partial pressure of carbon dioxide was monitored and maintained between 35 and 45 mmHg by manual ventilation at a rate of 8–12 breaths per minute. The anesthetized dogs were placed in left lateral recumbency and the right lateral neck region was clipped, prepared aseptically, and draped. An ~5.0-cm surgical cutdown was performed over the right jugular furrow to exteriorize the right jugular vein. Then an 8-Fr multipurpose catheter (Atom Medical Corp., Tokyo, Japan) was placed in the main pulmonary artery under fluoroscopic guidance. The right side of the neck was sutured, and all the dogs completely recovered from anesthesia through the conventional method (15).

### Creating Model Dogs With Chronic PH and Hemodynamic Measurements

The PAP was measured using circulatory function analysis software (SBP2000, Softron, Tokyo, Japan). The conscious dogs were restrained in the most stable position, and the PAP (systolic, mean, and diastolic) was measured invasively by calibrating with the atmospheric pressure. The average value of PAP calculated from nine consecutive cardiac cycles was considered as “baseline” data and used for the statistical analysis. After baseline PAP measurements were taken, microspheres measuring between 150 and 300 μm in diameter (Sephadex G-25 Coarse, Cytiva, Tokyo, Japan) were injected repeatedly and the peripheral pulmonary artery was embolized via the prepared catheter (16, 17). The time points at which systolic PAP (sPAP) rose to ~30, 40, and 50 mmHg were defined as “sPAP30,” “sPAP40,” and “sPAP50,” respectively. Each time point was at least 2 days after injection of the microspheres to eliminate the acute effects of microspheres on RV function. When the sPAP was maintained at 50 mmHg or more for 4 weeks without injection of microspheres, the time point was defined as “chronic” and the same examinations as those carried out at the other time points were performed. At each time point, the systemic arterial pressure was measured for all dogs using the oscillometric method. The dogs were sedated using butorphanol tartrate (0.1 mg/kg, IV) and midazolam hydrochloride (0.1 mg/kg, IV) to perform the microsphere injection, PAP measurements, and echocardiography when necessary.

### Echocardiographic Assessment of the Right Heart

Echocardiography was performed in all dogs on the same day as the hemodynamic measurements were taken at all time points. Conventional 2D and Doppler examinations were performed by a single investigator (RS) using a Vivid 7 or Vivid E95 echocardiographic system (GE Healthcare, Tokyo, Japan) and a 3.5–6.9 MHz transducer. A lead II electrocardiogram was recorded simultaneously and the images were displayed. Data obtained from at least five consecutive cardiac cycles in sinus rhythm from the dogs that were manually restrained in right and left lateral recumbency were stored. The images were analyzed using an offline workstation (EchoPAC PC, Version 204; GE Healthcare, Tokyo, Japan) by a single observer (YY).

For studying the right heart morphology, the end-diastolic and end-systolic RV areas (RVEDA and RVESA) along with the end-diastolic RV wall thickness (RVWTd) were measured using the left apical four-chamber view optimized for the right heart (RV focus view), as described previously (18–20). Each variable except for the RVWTd was measured by tracing the endocardial border of the right ventricle and normalized by body weight (20).

$$\text{RVEDA index=}\frac{\left(\text{RVEDA }[{\text{cm}}^{\text{2}}]\right)}{{\left(\text{body weight }[\text{kg}]\right)}^{\text{0}.\text{624}}}$$

$$\text{RVESA index=}\frac{\text{RVESA }({\text{cm}}^{\text{2}})}{\text{body weight }{\left(\text{kg}\right)}^{\text{0}.\text{628}}}$$

The RVWTd was measured as the largest diameter of the RV free wall at end-diastole using the B-mode method. Additionally, the ratio of pulmonary artery to aortic diameter (PA:Ao) was obtained from the right parasternal short-axis view at the level of the pulmonary artery, as described previously (21).

Tricuspid annular plane systolic excursion (TAPSE), RV fractional area change, peak systolic change (RV FAC), tissue Doppler imaging-derived peak systolic myocardial velocity of lateral tricuspid annulus (RV s'), RV myocardial performance index (RV MPI), RV stroke volume (RV SV), and RV cardiac output (RV CO) were measured as indicators of RV systolic function, as described previously (19, 20, 22). All RV functional variables were obtained from the RV focus view. The TAPSE was measured using the B-mode method as described previously (23–25). The TAPSE and RV FAC were normalized by body weight using the following formula (22, 25):

$$\text{TAPSEn=}\frac{\left(\text{TAPSE }[\text{cm}]\right)}{{\left(\text{body weight }[\text{kg}]\right)}^{\text{0}.\text{284}}}$$

$$\text{RV FACn=}\frac{\left(\text{RV FAC }[\%]\right)}{{\left(\text{body weight }[\text{kg}]\right)}^{\text{-0}.\text{097}}}$$

The RV MPI was obtained from the tissue Doppler imaging-derived lateral tricuspid annular motion wave using the following formula:

$$\begin{array}{l} RV\;MPI=\frac{\left(b-a\right)}{a} \end{array}$$

where a is the duration of the systolic tricuspid annular motion wave, and b is the interval from the end of the late diastolic tricuspid annular motion wave to the onset of the early diastolic tricuspid annular motion wave (19). The RV SV was calculated by multiplying the velocity-time integral of the pulmonary artery flow and the cross-sectional area of the pulmonary trunk obtained from the right parasternal short-axis view at the level of pulmonary artery, as described previously (26). The RV CO was obtained using RV SV and heart rate calculated by mean R-R intervals obtained from the same cardiac cycle used for RV SV measurement.

If the dogs had tricuspid valve or pulmonary valve regurgitation, we classified these severities as mild, moderate, or severe using color Doppler and continuous wave Doppler methods, as described previously (27, 28).

### Two-Dimensional Speckle Tracking Echocardiography

**Figure 1:**
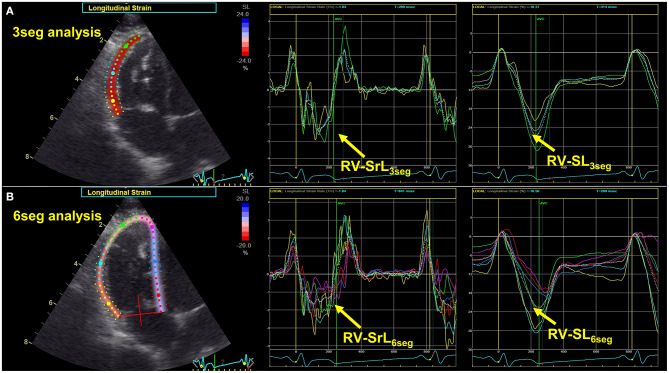
Two-dimensional speckle tracking echocardiography-derived right ventricular longitudinal strain and strain rate (RV-SL and RV-SrL, respectively). **(A)** RV-SL and RV-SrL of the right ventricular free wall (RV-SL_3seg_ and RV-SrL_3seg_, respectively). **(B)** RV-SL and RV-SrL of the global right ventricle (RV-SL_6seg_ and RV-SrL_6seg_, respectively).

All 2D-STE analyses were performed by a single investigator using the same ultrasound machine and evaluated by the same investigator using the same offline workstation as that used for standard echocardiography. The strain and strain rate were obtained from the RV focus view using the left ventricular four-chamber algorithms (23, 29). The region of interest for 2D-STE was defined by manually tracing the RV endocardial border. Only RV free wall analysis (3seg) was performed by tracing from the level of the lateral tricuspid annulus to the RV apex for the longitudinal strain (RV-SL_3seg_) and strain rate (RV-SrL_3seg_) (Figure 1A). Right ventricular global analysis (6seg) was also performed by tracing from the lateral tricuspid annulus to the septal tricuspid annulus (including the interventricular septum) *via* the RV apex for the 6seg longitudinal strain (RV-SL_6seg_), and strain rate (RV-SrL_6seg_) (Figure 1B). Manual adjustments were made to include and track the entire myocardial thickness over the cardiac cycle when necessary. When the automated software could not track the myocardial regions, the regions of interest were retraced and recalculated. The RV-SL was defined as the absolute value of the negative peak value obtained from the strain wave (23, 30). The RV-SrL was obtained from the strain rate wave and was defined as the absolute value of the negative peak value during systole (30–32).

### Variability of Intra- and Inter-Observer Measurements

Intra-observer measurement of variability was performed by a single observer who performed all the echocardiographic and radiographic measurements (YY). The baseline RV morphological and functional indices were obtained from the seven dogs. All measurements were performed on two different days at >7-day intervals using the same cardiogram and cardiac cycles. A second blinded observer (HK) measured the same indices for the determination of inter-observer variability using the same echocardiogram and heart cycles.

### Statistical Analysis

All statistical analyses were performed using the commercially available EZR software, version 1.41 (Saitama Medical Center, Jichi Medical University, Saitama, Japan) (33). All continuous data were reported as median (interquartile range).

The normality of data was tested using the Shapiro–Wilk test. Continuous variables were compared between each timepoint by means of repeated measures analysis of variance with subsequent pairwise comparisons using the Bonferroni-adjusted paired *t*-test for normally distributed data or Friedman rank sum test with subsequent pairwise comparisons using the Bonferroni-adjusted Wilcoxon signed rank sum test for non-normally distributed data.

Variability of intra- and inter-observer measurements was quantified by the coefficient of variation (CV), which was calculated using the following formula:

$$\begin{array}{l} CV\;\left(\%\right)=\frac{\left(standard\;deviation\right)}{\left(mean\;value\right)}\times 100 \end{array}$$

Intra- and inter-class correlation coefficients (ICC) were also used to evaluate the measurement variability. Low measurement variability was defined as CV <10.0 and ICC > 0.7. Statistical significance was set at *P* <0.050.

## Results

### Creating PH Model Dogs

The dogs were administered repeated microsphere infusions for 2.2 ± 1.0, 6.9 ± 3.8, 14.9 ± 5.7, and 50.9 ± 13.1 weeks to meet the definition of sPAP30, sPAP40, sPAP50, and chronic, respectively. The median total dose of microspheres was 1.24 mg/kg (range: 0.93–1.37). There was no significant change in body weight. Two dogs required sedation with butorphanol tartrate and midazolam hydrochloride to perform echocardiography and for taking PAP measurements at each timepoint. None of the dogs showed any clinical symptoms associated with PH, including syncope, dyspnea, lethargy, ascites, and pleural effusion throughout this study protocol.

### Hemodynamic Measurements

The hemodynamic data obtained from all seven model dogs were included in the statistical analysis. Table 1 shows the results of hemodynamic parameters in the PH model dogs. With the rise in sPAP, the mean PAP also increased in the sPAP30, sPAP40, sPAP50, and chronic phases compared with the baseline (*P* = 0.012, *P* = 0.018, *P* <0.001, and *P* = 0.021, respectively) parameters. The diastolic PAP was significantly increased in the sPAP50 and chronic phases compared with the baseline and sPAP30 values (sPAP50: *P* = 0.003 and *P* = 0.004, respectively; chronic: *P* = 0.013 and *P* = 0.023, respectively). There were no significant changes in systolic, mean, and diastolic systemic arterial pressure and heart rate with increased sPAP.

**Table 1:** Changes in hemodynamic parameters during the process of creating canine models of chronic embolic pulmonary hypertension.

**Variables**	**Baseline**	**sPAP30**	**sPAP40**	**sPAP50**	**Chronic**
**Pulmonary arterial pressure (mmHg)**
Systole	20.0 (17.3, 24.6)	33.0 (30.0, 34.6)^a^	42.3 (40.4, 47.8)^a^^b^	52.4 (50.7, 52.9)^a^^b^^c^	51.4 (50.3, 65.9)^a^^b^^c^
Mean	12.8 (11.0, 15.0)	16.8 (16, 20.4)^a^	21.7 (18.3, 23.9)^a^^b^	29.4 (27.9, 33.7)^a^^b^^c^	30.1 (29.3, 31.9)^a^^b^^c^
Diastole	6.4 (5.1, 9.0)	8.8 (8.3, 12.6)	11.8 (7, 17.2)	16.1 (15.4, 18.8)^a^^b^	16.3 (15.2, 19.4)^a^^b^
**Systemic arterial pressure (mmHg)**
Systole	126 (116, 134)	124 (113, 137)	132 (116, 136)	130 (130, 131)	128 (120, 142)
Mean	92 (88, 97)	91 (78, 106)	97 (81, 107)	99 (91, 102)	93 (82, 98)
Diastole	80 (70, 82)	75 (65, 87)	82 (64, 93)	80 (73, 88)	69 (60, 77)
Heart rate (bpm)	88 (81, 115)	99 (91, 120)	102 (82, 124)	94 (64, 112)	84 (81, 100)

*Continuous variables were displayed as median (interquartile range)*.

a *The value is significantly different from the Baseline (P < 0.050)*.

b *The value is significantly different from the sPAP30 (P < 0.050)*.

c *The value is significantly different from the sPAP40 (P < 0.050)*.

### Echocardiographic Measurements

In this study, the echocardiographic data obtained from all seven model dogs were included in the statistical analysis. All the dogs had mild pulmonary valve regurgitation at baseline, sPAP30, and sPAP40 phases, and that was progressed to moderate at sPAP50 and chronic phases in four dogs (57%). Additionally, three dogs (43%) had mild tricuspid valve regurgitation at each timepoint.

Table 2 shows the results of echocardiographic parameters for RV morphology and function. The PA:Ao value was significantly higher in the sPAP50 phase than the baseline values and those of the sPAP40 phase (*P* = 0.034 and *P* = 0.038, respectively). This value was also significantly elevated in the chronic phase compared with those in the baseline, sPAP30, and sPAP40 phases (*P* = 0.021, *P* = 0.004, and *P* = 0.010, respectively). The RVEDA index and RVESA index were significantly increased in the chronic phase compared with those in the sPAP30 phase (*P* = 0.041 and *P* = 0.048, respectively). The RVWTd was significantly higher in the sPAP50 phase compared with that in baseline phase (*P* = 0.042). This value was also significantly elevated in the chronic phase compared with those in the baseline and sPAP30 phases (*P* = 0.002 and *P* = 0.047, respectively). The TAPSEn and RV MPI were significantly worse in the chronic phase compared with the baseline values (*P* = 0.008 and *P* = 0.003, respectively). The RV SV was significantly higher in the chronic phase compared with that in the sPAP30 phase (*P* = 0.016), whereas, RV FACn, RV s', and RV CO showed no significant changes with increased sPAP.

**Table 2:** Changes in echocardiographic parameters during the process of creating canine models of chronic embolic pulmonary hypertension.

**Variables**	**Baseline**	**sPAP30**	**sPAP40**	**sPAP50**	**Chronic**
PA:Ao	0.80 (0.78, 0.81)	0.78 (0.76, 0.81)	0.78 (0.74, 0.79)	0.92 (0.85, 0.93)^a^^c^	0.97 (0.96, 0.99)^a^^b^^c^
RVEDA index (cm^2^/kg^0.624^)	1.43 (1.11, 1.44)	1.08 (0.91, 1.17)	1.02 (0.96, 1.27)	1.08 (0.96, 1.14)	1.47 (1.28, 1.63)^b^
RVESA index (cm^2^/kg^0.628^)	0.77 (0.60, 0.85)	0.73 (0.55, 0.82)	0.72 (0.52, 0.82)	0.65 (0.60, 0.72)	0.99 (0.88, 1.10)^b^
RVWTd (mm)	3.7 (3.4, 3.8)	4.0 (3.8, 4.2)	4.4 (3.9, 4.9)	4.9 (4.5, 5.0)^a^	5.6 (5.2, 6.1)^a^^b^
RV FACn (%/kg^−0.097^)	53.7 (49.1, 61.0)	47.9 (46.3, 50.2)	44.0 (42.7, 56.6)	47.7 (41.6, 50.1)	37.1 (36.7, 40.0)
TAPSEn (mm/kg^0.33^)	6.2 (5.9, 6.6)	5.5 (3.9, 5.9)	6.2 (6.0, 6.6)	5.9 (5.3, 6.4)	4.6 (4.3, 5.5)^a^
RV s' (cm/s)	11.0 (10.6, 12.2)	11.8 (9.8, 14.0)	13.1 (11.6, 14.8)	13.4 (11.6, 14.7)	9.1 (7.6, 11.0)
RV MPI	0.41 (0.36, 0.43)	0.41 (0.39, 0.56)	0.55 (0.43, 0.64)	0.55 (0.44, 0.62)	0.72 (0.68, 0.76)^a^
RV SV (mL)	18.5 (16.1, 19.4)	15.0 (10.9, 16.6)	15.5 (13.4, 17.6)	16.9 (15.7, 21.7)	21.8 (20.8, 23.4)^b^
RV CO (L/min)	1.5 (1.3, 2.2)	1.5 (1.4, 1.6)	1.5 (1.3, 1.9)	1.5 (1.4, 1.6)	1.8 (1.7, 2.1)

*PA:Ao, pulmonary artery to aortic diameter ratio; RV CO, right ventricular cardiac output; RV FACn, right ventricular fractional area change normalized by body weight; RV MPI, right ventricular myocardial performance index; RV s', tissue Doppler imaging-derived peak systolic myocardial velocity of lateral tricuspid annulus; RV SV, right ventricular stroke volume; RVEDA index, end-diastolic right ventricular area normalized by body weight; RVESA index, end-systolic right ventricular area normalized by body weight; RVWTd, end-diastolic right ventricular wall thickness; TAPSEn, tricuspid annular plane systolic excursion normalized by body weight*.

*Continuous variables were displayed as median (interquartile range)*.

a *The value is significantly different from Baseline (P < 0.050)*.

b *The value is significantly different from sPAP30 (P < 0.050)*.

c *The value is significantly different from sPAP40 (P < 0.050)*.

**Figure 2:**
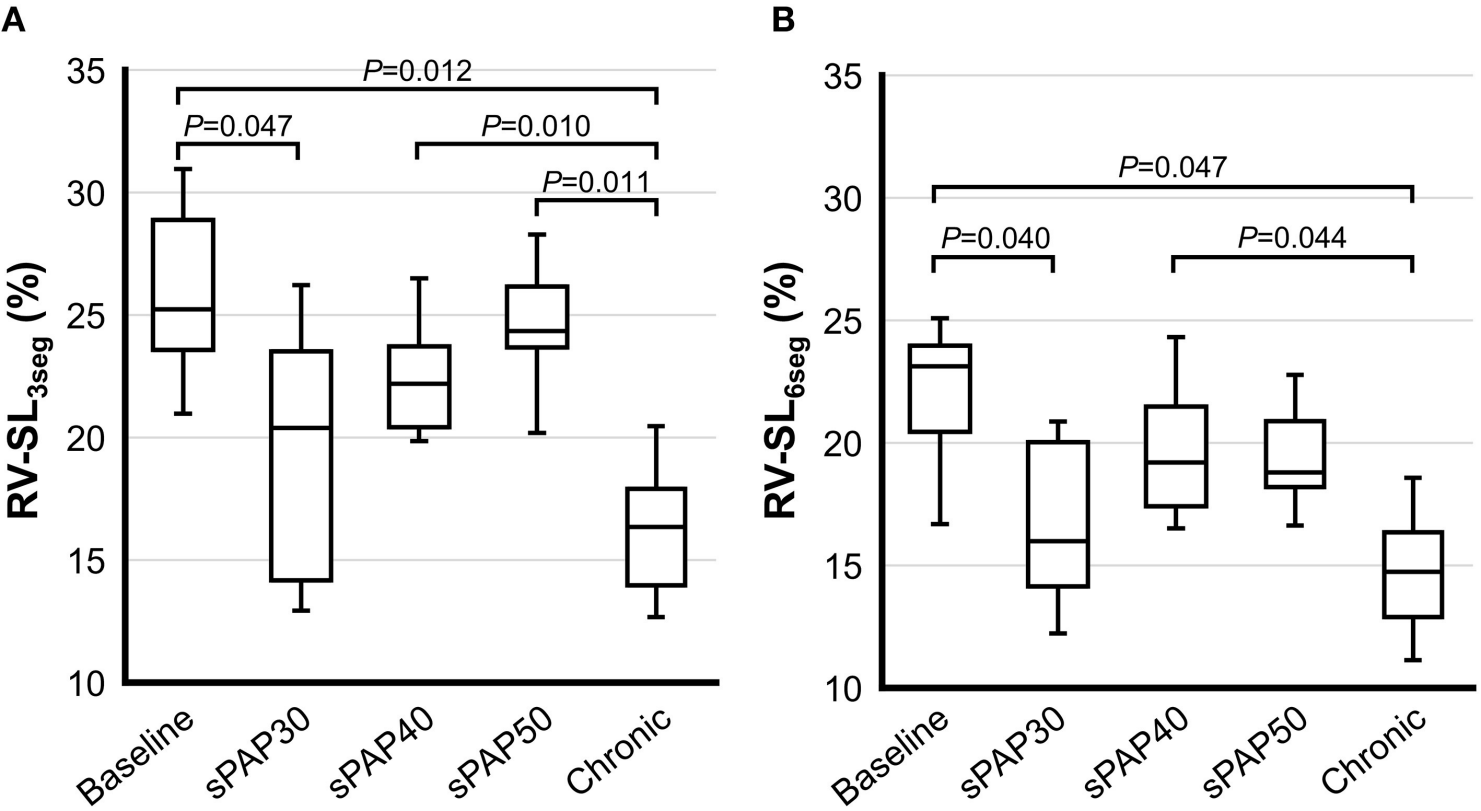
Box and whisker plots of the right ventricular longitudinal strain (RV-SL) obtained from two-dimensional speckle tracking echocardiography. The bottom of the box is 25%, the middle line is the median, the top of the box is 75%, and the whiskers represent the range. **(A)** RV-SL of the right ventricular free wall (RV-SL_3seg_). **(B)** RV-SL of the global right ventricle (RV-SL_6seg_).

**Figure 3:**
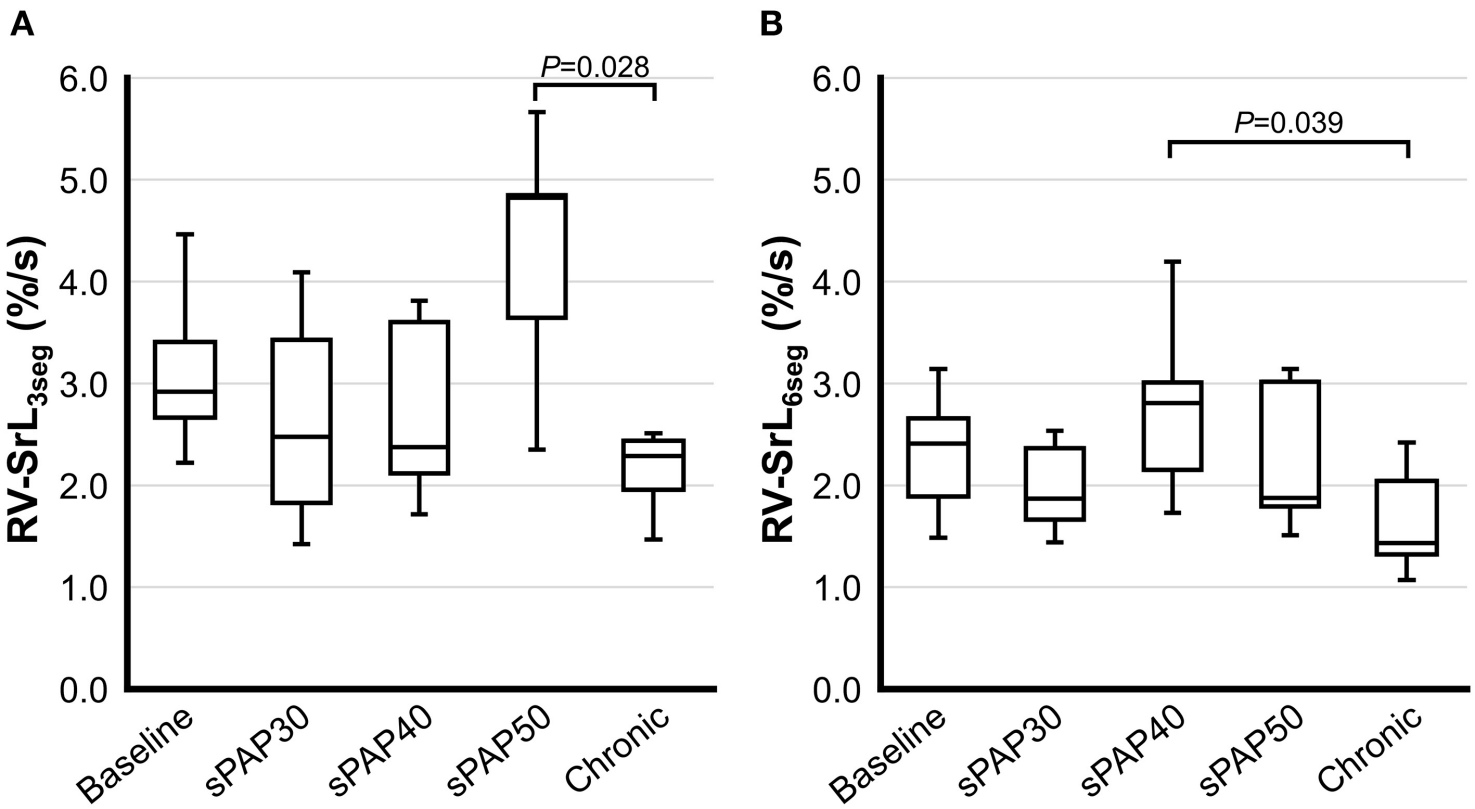
Box and whisker plots of the right ventricular longitudinal strain rate (RV-SrL) obtained from two-dimensional speckle tracking echocardiography. The bottom of the box is 25%, the middle line is the median, the top of the box is 75%, and the whiskers represent the range. **(A)** RV-SrL of the right ventricular free wall (RV-SrL_3seg_). **(B)** RV-SrL of the global right ventricle (RV-SrL_6seg_).

The results of 2D-STE indices are summarized in Figures 2, 3. The RV-SL_3seg_ and RV-SL_6seg_ were significantly decreased in the sPAP30 phase compared with the baseline values (*P* = 0.047 and *P* = 0.040, respectively). Additionally, RV-SL_3seg_ was significantly lower in the chronic phase compared with those in the baseline, sPAP40, and sPAP50 phases (*P* = 0.012, *P* = 0.010, and *P* = 0.011, respectively) (Figure 2A). However, RV-SL_6seg_ was significantly reduced in the chronic phase compared with those in the baseline and sPAP40 phases (*P* = 0.047 and *P* = 0.044, respectively) (Figure 2B). The RV-SrL_3seg_ and RV-SrL_6seg_ were significantly lower in the chronic phase compared with those in the sPAP50 and sPAP40 phases, respectively (*P* = 0.028 and *P* = 0.039, respectively) (Figure 3).

### Intra- and Inter-Observer Measurement Variability

The results of intra- and inter-observer measurement variability for the echocardiographic indices assessed in this study are summarized in Table 3. Considering the intra-observer measurement variability, all the echocardiographic parameters showed low measurement variability. Further, all the echocardiographic indices except for RVESA, RV FAC, and RV MPI showed low measurement variability based on CV and ICC.

**Table 3:** Inter- and intra-observer measurement variability for echocardiographic parameters evaluated in this study.

**Variables**	**Intra-observer**			**Inter-observer**		
	**CV (%)**	**ICC**	***P***	**CV (%)**	**ICC**	***P***
PA:Ao	4.2	0.94	<0.001	5.9	0.80	0.012
RVEDA	4.1	0.99	<0.001	8.6	0.82	<0.001
RVESA	5.8	0.94	<0.001	11.4	0.66	0.012
RVWTd	3.2	0.93	<0.001	6.5	0.80	0.004
RV FAC	5.0	0.81	0.001	8.8	0.50	0.002
TAPSE	3.1	0.90	<0.001	5.9	0.80	0.002
RV s'	2.8	0.97	<0.001	2.9	0.95	<0.001
RV MPI	7.8	0.88	<0.001	12.3	0.64	0.002
RV SV	4.4	0.98	<0.001	8.9	0.94	<0.001
RV-SL_3seg_	4.4	0.93	<0.001	5.0	0.92	<0.001
RV-SrL_3seg_	6.1	0.95	<0.001	9.3	0.89	<0.001
RV-SL_6seg_	5.2	0.90	<0.001	7.2	0.85	<0.001
RV-SrL_6seg_	5.8	0.94	<0.001	7.6	0.93	<0.001

*3seg, only right ventricular free wall analysis; 6seg, right ventricular global analysis; CI, confidence interval; CV, coefficient of variation; ICC, intra- or inter-class correlation coefficients; PA:Ao, pulmonary artery to aortic diameter ratio; RV FAC, right ventricular fractional area change; RV MPI, right ventricular myocardial performance index; RV s', tissue Doppler imaging-derived peak systolic myocardial velocity of lateral tricuspid annulus; RV SV, right ventricular stroke volume; RVEDA, end-diastolic right ventricular area; RVESA, end-systolic right ventricular area; RV-SL, right ventricular longitudinal strain; RV-SrL, right ventricular longitudinal strain rate; RVWTd, end-diastolic right ventricular wall thickness; TAPSE, tricuspid annular plane systolic excursion*.

## Discussion

We created model dogs with chronic pre-capillary PH with moderately increased PAP and substantial right heart remodeling and compared the changes in RV function associated with increased PAP that was measured invasively in conscious dogs. In the acute phase, 2D-STE-derived RV systolic function was temporarily decreased due to the acute rise in PAP; however, it improved with RV hypertrophy; this may be a sign of RV adaptive remodeling. In contrast to the acute phase, RV systolic dysfunction assessed by RV-SL and RV dilatation were observed in the chronic phase of PH, which could be because of RV maladaptive remodeling and myocardial dysfunction.

In this study, certain conventional echocardiographic indices, such as TAPSEn and RV MPI, did not significantly change with increased PAP in the acute phase, although these indices were significantly worse in the chronic phase of PH. Nonetheless, 2D-STE indices showed substantial changes in the acute phase as well as in the chronic phase of PH. Previous studies reported that these conventional echocardiographic indices are affected by angle- and load-dependent limitations (34–36). Furthermore, all the model dogs in our study had echocardiographic evidence of tricuspid valve and pulmonary valve regurgitation; therefore, these volume overloads may prevent the detection of RV dysfunction using conventional indices. In contrast, 2D-STE variables have been used to assess intrinsic RV myocardial function with angle-independency and the low effect of these loading conditions (10). Additionally, considering PH would induce full RV remodeling against RV pressure overload, assessment of the global right ventricular function based on 2D-STE indices may help detect precise RV myocardial function more sensitively than conventional indices. Therefore, 2D-STE indices can be used to detect changes in intrinsic RV systolic function, which cannot be detected using conventional indices.

In the acute phase, RV-SL was significantly reduced in the sPAP30 phase and gradually increased in the sPAP40 and sPAP50 phases. In the sPAP30 phase, all the dogs did not have RV remodeling; therefore, the acute rise in PAP might have induced the imbalance between RV contractility and RV pressure overload (i.e., RV arterial uncoupling caused by increased RV pressure overload). However, RV-SL gradually increased with PAP and RVWTd, which indicates RV adaptive remodeling. In human medicine, RV adaptation remodeling was induced in PH patients through various mechanisms such as neurological activation, inflammation, and altered bioenergetics (19, 37, 38). In this study, the RVWTd gradually increased with the rise in PAP during the process of creating the chronic PH model dogs. Therefore, our results indicate that 2D-STE indices may be highly sensitive to changes that reflect the adaptation in RV myocardial contractility.

There was a significant difference between the 2D-STE-derived RV systolic functional indices of the sPAP50 and the chronic phases, although there was no significant difference in the RV loading condition (sPAP was stable at 50 mmHg). In general, chronic RV pressure overload increases myocardial wall stress, which in turn increases RV wall thickness and contractility to maintain RV CO (6–9). However, the RV myocardial contractility may be unable to cope with the chronic, excessive pressure overload. Our results suggest that RV-SL may reflect the intrinsic RV myocardial contractility, which showed decompensation in the chronic phase. Additionally, myocardial fibrosis might have also affected the results. Several studies have reported the prevalence of RV myocardial fibrosis in patients with PH (39, 40). Furthermore, in human patients with severe heart failure, the 2D-STE-derived RV-SL was reported to have a strong correlation with RV myocardial fibrosis, which could induce RV maladaptive remodeling and subsequent RV myocardial dysfunction (41). Although we have not conducted histopathological examinations in all the model dogs, the changes in RV-SL may also be indicative of RV fibrosis and RV maladaptation with PH progression.

In this study, RV-SL changed more drastically in 3seg than in 6seg in the acute phase, although both indices were significantly decreased in the chronic phase. Considering the interventricular septum when evaluating RV function is a matter of controversy (14, 42). A previous study has reported that RV-SL in the RV free wall is more sensitive to mild RV pressure overload than that in the interventricular septum (14). Our results also suggest that 2D-STE indices in the RV free wall may be more sensitive to increased RV pressure overload than those in the interventricular septum in dogs with moderate RV pressure overload. However, through the current 2D-STE assessment, we could not distinguish between the interventricular septum function of the RV and left ventricular components. Our results may vary in dogs with moderate PH secondary to left heart disease, which might impair the septal left ventricular function. Further studies to assess the precise myocardial function of both ventricles in dogs with PH secondary to left heart disease are expected in the future.

Our study has several limitations. First, the results were obtained from dogs with PH that was experimentally induced by microsphere infusion, which may vary in actual clinical settings or dogs with PH. Additionally, our findings may not be applicable in dogs with PH because of other causes such as left heart disease owing to its different pathophysiology that can increase PAP. Further studies are warranted to evaluate the relationship between echocardiographic indices and invasive PAP in dogs with spontaneous PH. Second, two of the seven dogs were sedated using butorphanol tartrate and midazolam hydrochloride. However, sedation with these agents have minimal effect on cardiovascular function (43). Furthermore, in dogs that required sedation so that hemodynamic measurements could be taken and echocardiography could be performed, these agents were used throughout the study protocol. Thus, sedation would have had minimal effect on our results. Finally, we have evaluated only longitudinal RV strain and strain rate. RV circumferential function would also contribute to RV systolic function in addition to the longitudinal function (30, 44, 45).

In conclusion, our study found that 2D-STE-derived RV-SL was significantly decreased in the sPAP30 phase compared with that in the baseline phase; it gradually increased in the sPAP40 and sPAP50 phases compared with that in the sPAP30 phase and decreased in the chronic phase compared with the baseline and sPAP50 phases. These results suggest that this non-invasive echocardiographic variable may reflect the RV compensative mechanism against PH pathophysiology, which could not be detected by conventional echocardiographic indices for RV function.

## Data Availability Statement

The raw data supporting the conclusions of this article will be made available by the authors, without undue reservation.

## Ethics Statement

The animal study was reviewed and approved by Ethical committee for laboratory animal use of the Nippon Veterinary and Life Science University.

## Author Contributions

YY performed the concept/design, data analysis/interpretation, drafting article, and critical revision of article. RS performed the concept/design, data analysis/interpretation, critical revision of article, and approved the article. HK performed the data analysis as the second observer. TT, HM, and HK performed data interpretation, critically revised the manuscript, and approved the article. All authors contributed to the article and approved the submitted version.

## Conflict of Interest

HK received a grant from Toray Industries, Inc. The remaining authors declare that the research was conducted in the absence of any commercial or financial relationships that could be construed as a potential conflict of interest.

## Publisher's Note

All claims expressed in this article are solely those of the authors and do not necessarily represent those of their affiliated organizations, or those of the publisher, the editors and the reviewers. Any product that may be evaluated in this article, or claim that may be made by its manufacturer, is not guaranteed or endorsed by the publisher.

## Abbreviations

2D-STE: two-dimensional speckle tracking echocardiography
3seg: only right ventricular free wall analysis
6seg: right ventricular global analysis
CV: coefficient of variation
PA:Ao: pulmonary artery to aortic diameter ratio
PAP: pulmonary arterial pressure
PH: pulmonary hypertension
RV CO: right ventricular cardiac output
RV FACn: right ventricular fractional area change normalized by body weight
RV MPI: right ventricular myocardial performance index
RV s': tissue Doppler imaging-derived peak systolic myocardial velocity of lateral tricuspid annulus
RV SV: right ventricular stroke volume
RVEDA index: end-diastolic right ventricular area normalized by body weight
RVESA index: end-systolic right ventricular area normalized by body weight
RV-SL: right ventricular longitudinal strain
RV-SrL: right ventricular longitudinal strain rate
RVWTd: end-diastolic right ventricular wall thickness
TAPSEn: tricuspid annular plane systolic excursion normalized by body weight.
